# 495. *De novo* emergence of resistance mutations during treatment of SARSCoV-2 infection in immunocompromised patients

**DOI:** 10.1093/ofid/ofad500.564

**Published:** 2023-11-27

**Authors:** Katherine Johnson, Rosemary Soave, Wei Wang, Joshua Albert, Rosy Priya Kodiyanplakkal, Maria Salpietro, Hsu Jing-Mei, Diego G Diel, Elodie Ghedin, Mirella Salvatore

**Affiliations:** NIAID/NIH, Bethesda, Maryland; NIAID/NIH, Bethesda, Maryland; NIAID/NIH, Bethesda, Maryland; Weill Cornell Medicine, NewYork-Presbyterian Hospital, New York, New York; Weill Cornell Medicine, New York, New York; NYU, New York, New York; Cornell University, Ithaca, New York; National Institute of Allergy and Infectious Diseases (NIAID)/National Institutes of Health (NIH), MD; Weill Cornell Medical College, new york, New York

## Abstract

**Background:**

Vaccination and therapeutic strategies for COVID-19 are constantly hindered by the rapid emergence of new viral variants less sensitive to antibodies or antivirals. Emerging evidence points to prolonged replication in the immunocompromised host as a key factor contributing to emergence of viral genomic mutations. We analyzed viral evolution and emergence of intra-host variants in response to treatments including antivirals and monoclonal antibodies in immunocompromised subjects with hematological malignancies infected with SARS-CoV2.

**Methods:**

From April 2020 to August 2022, we collected 245 nasopharyngeal samples from 92 patients including longitudinal samples of 31 subjects with persistent infection (detection of SARS-CoV-2 RNA ≥30 days regardless of symptomatology). The number of samples per subject ranged from 1-14. To identify SARS-CoV-2 mutations, we performed whole genome sequencing using an amplicon-based approach and the Illumina NextSeq platform. Only samples that had 75% genome covered at 5x read depth were included in the analysis.

**Results:**

After quality control filtering, 21 subjects had at least 2 samples available for longitudinal analyses. Ten of them had no consensus changes during infection (compared to the first sample collected), and 11 had at least 1 change. The spike protein was the most likely location for a consensus change (9 patients), followed by consensus changes in nsp3, nsp12, nsp13, N, ORF8, and nsp4. Mutations tended to appear mostly following treatment, with an increased number of changes apparently associated with worse outcome. After treatment, 3 patients developed mutations in the RNA-dependent RNA polymerase (nsp12) that are known to be associated with remdesivir resistance. One Paxlovid treated patient developed a nsp5 protease mutation potentially associated with resistance. Another patient developed a spike mutation associated with resistance to sotrovimab.

**Conclusion:**

Our results underscore the need for augmented efforts to study intra-host evolution in the immunocompromised host, identify and isolate viruses resistant to treatment(s) and identify host determinants associated with accelerated resistance. These results are important for implementing optimal treatment strategies and to inform infection control measures.

**Disclosures:**

**All Authors**: No reported disclosures

